# Supplementary material for: Synergistic Effect of Methyl Jasmonate and Abscisic Acid Co-Treatment on Avenanthramide Production in Germinating Oats
Source: Int J Mol Sci. 2021 Apr 30;22(9):4779. doi: 10.3390/ijms22094779 (PMC8125723; doi:10.3390/ijms22094779)
Supplement: Supplementary file 1 [file ijms-22-04779-s001.zip › ijms-1201230-supplementary.pdf]

Supporting Information

Supplementary data

Table S1 Primer pairs used for qRT-PCR in this study.

Gene	Gene information	Primers sequence
AsActin	KP257585	Forward : 5'- GCTGTGCTTTCCCTCTATGC -3'
		Reverse : 5'- GGACATCAAGGAGAAGCTCG -3'
AsPAL	MH507022	Forward : 5'- GACAACCCGCTCATTGACGT -3'
		Reverse : 5'- ATCTCAGCACCCTTGAAGCC -3'
As4CL	MH397063	Forward : 5'- CAGAGGCCACAAAGAACACG -3'
		Reverse : 5'- GCCGGAGGAACTTGGAATCC-3'
AsCCoAOMT	MK577959	Forward : 5'- CATGGAGATCGGCGTGTACA -3'
		Reverse : 5'- TAGTTGTCGCGGTTGATGTC -3'
AsHHT1	AB076980	Forward : 5'- AATGAACAAACAGCACGCCC -3'
		Reverse : 5'- TTGCAGTAGTAGCTACGTCTGG -3'
AsHHT2	AB076981	Forward : 5'- TCGCCTTCGTGCTCCCCAG -3'
		Reverse : 5'- GCGCGTGCTGTTTGTTCGTT -3'
AsHHT3	AB076982	Forward : 5'- CGGCGACGCGTGCTGCTA -3'
		Reverse : 5'- CACCCCCTCAGCGTTGCAG -3'
AsHHT4	MH397064	Forward : 5'- CTGGAGCCGGAGCGGGA -3'
		Reverse : 5'- CGACGAAGAGCACCCCCTC -3'
AsHHT5	MH397065	Forward : 5'- GGGCGCTGTGGCAGATGGAG -3'
		Reverse : 5'- TCCCCAACCGAAATCCGCGTT -3'
AsHHT6	MH397066	Forward : 5'- GAGCCGGAGCAGGGGAGG -3'
		Reverse : 5'- CGACGAAGAGCACCCCCTC -3'

PAL, phenylalanine ammonia lyase; 4CL, 4-coumarate-CoA ligase; CCoAOMT, caffeoyl-CoA O-methyltransferase; HHT, hydroxycinnamoyl-CoA:hydroxyanthranilate N-hydroxycinnamoyl transferase.

Primers were designed using the Beacon designer™ software (Premier Biosoft).

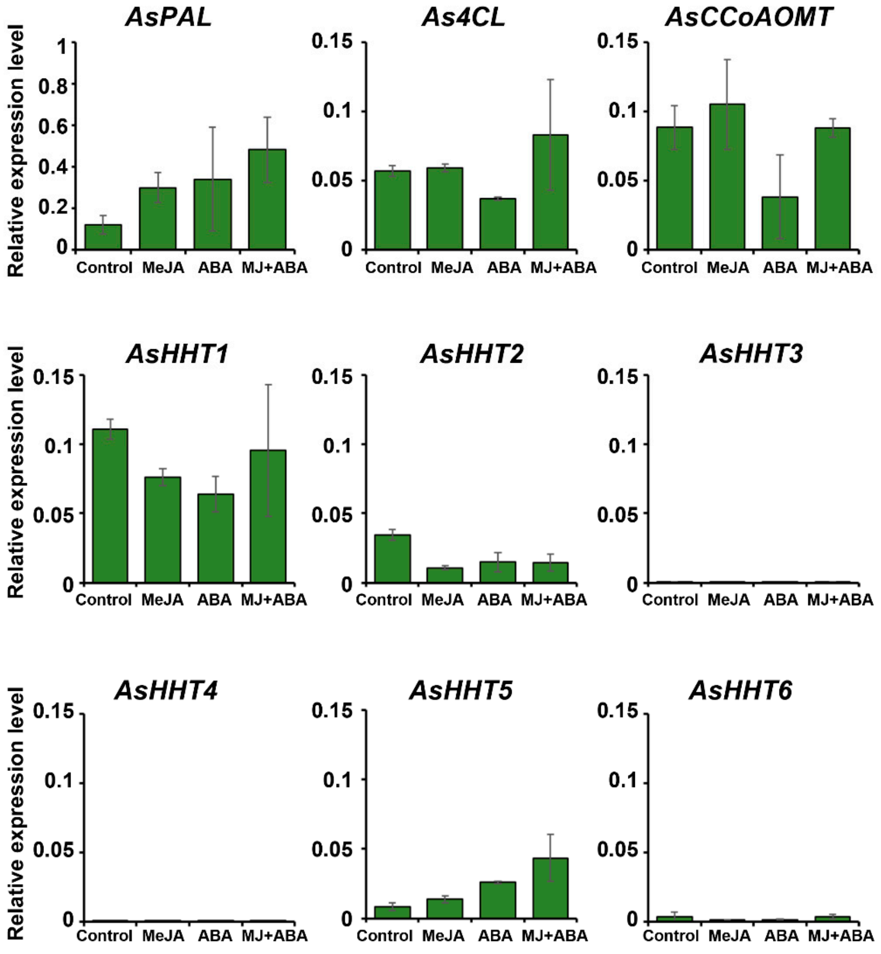

Figure S1. The relative expression of avenanthramide-biosynthetic genes following co-treatment with MeJA and ABA of germinating oats. Transcript levels of *PAL, 4CL, CCoAOMT, HHT1, HHT2, HHT3, HHT4, HHT5*, and *HHT6* were analyzed by qRT-PCR in germinating oats treated with 75 μM MeJA and 25 μM ABA for 3 days. Relative expression levels were normalized against *AsActin* (KP257585) and are presented as fold induction relative to *AsActin*. Data represent the mean of three independent replicates ± SD.

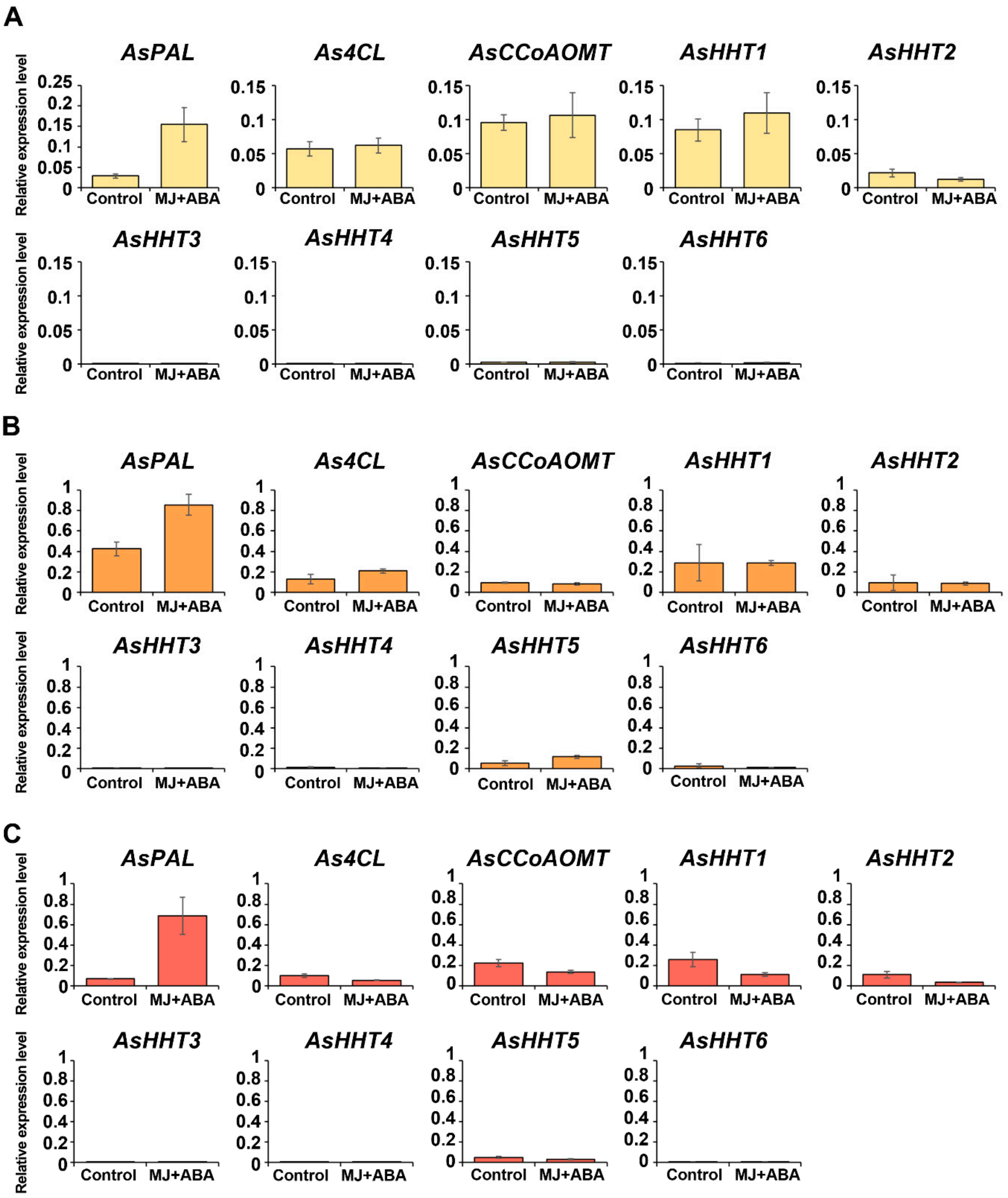

Figure S2. The relative expression levels of avenanthramide-biosynthetic genes in three types of oat tissues: (A) leaves, (B) grains, and (C) roots. Combined treatment (MeJA + ABA) comprised 75 µM MeJA and 25 µM ABA. Two-day-old germinating oats were treated for 3 days, and transcript levels of *PAL, 4CL, CCoAOMT, HHT1, HHT2, HHT3, HHT4, HHT5*, and *HHT6* were analyzed by qRT-PCR. Relative expression levels were normalized against *AsActin*

(KP257585) and are presented as fold induction relative to the control. Data represent the mean of three independent replicates ± SD.
